# Total hip arthroplasties in the Dutch Arthroplasty Register (LROI) and the Nordic Arthroplasty Register Association (NARA): comparison of patient and procedure characteristics in 475,685 cases

**DOI:** 10.1080/17453674.2020.1843875

**Published:** 2020-11-10

**Authors:** Liza N Van Steenbergen, Keijo T Mäkelä, Johan Kärrholm, Ola Rolfson, Søren Overgaard, Ove Furnes, Alma B Pedersen, Antti Eskelinen, Geir Hallan, Berend W Schreurs, Rob G H H Nelissen

**Affiliations:** a Dutch Arthroplasty Register (LROI), ‘s- Hertogenbosch, the Netherlands;; b Department of Orthopaedics and Traumatology, Turku University Hospital, Turku, Finland;; c The Finnish Arthroplasty Register, Helsinki, Finland;; d Department of Orthopaedics, Sahlgrenska University Hospital, Gothenburg, Sweden;; e Institute of Clinical Sciences, Sahlgrenska Academy, University of Gothenburg, Gothenburg, Sweden;; f The Swedish Hip Arthroplasty Register, Gothenburg, Sweden;; g Department of Orthopaedic Surgery and Traumatology, Odense University Hospital, Odense, Denmark;; h Department of Clinical Research, University of Southern Denmark, Odense, Denmark;; i The Danish Hip Arthroplasty Register, Aarhus, Denmark;; j The Norwegian Arthroplasty Register, Department of Orthopaedic Surgery, Haukeland University Hospital, Bergen, Norway;; k Department of Clinical Medicine, Faculty of Medicine, University of Bergen, Bergen, Norway;; l Department of Clinical Epidemiology, Aarhus University Hospital, Aarhus, Denmark;; m Coxa Hospital for Joint Replacement, and Faculty of Medicine and Health Technologies, University of Tampere, Tampere, Finland;; n Department of Orthopaedics, Radboudumc, Nijmegen, the Netherlands;; o Department of Orthopaedics, Leiden University Medical Centre, Leiden, the Netherlands

## Abstract

Background and purpose — Collaborations between arthroplasty registries are important in order to create the possibility of detecting inferior implants early and improve our understanding of differences between nations in terms of indications and outcomes. In this registry study we compared patient and procedure characteristics, and revision rates in the Nordic Arthroplasty Register Association (NARA) database and the Dutch Arthroplasty Register (LROI).

Patients and methods — All total hip arthroplasties (THAs) performed in 2010–2016 were included from the LROI (n = 184,862) and the NARA database (n = 290,823), which contains data from Denmark, Norway, Sweden, and Finland. Descriptive statistics and Kaplan–Meier survival analyses based on all reasons for revision and stratified by fixation were performed and compared between countries.

Results — In the Netherlands, the proportion of patients aged < 55 years (9%) and male patients (34%) was lower than in Nordic countries (< 55 years 11–13%; males 35–43%); the proportion of osteoarthritis (OA) (87%) was higher compared with Sweden (81%), Norway (77%), and Denmark (81%) but comparable to Finland (86%). Uncemented fixation was used in 62% of patients in the Netherlands, in 70% of patients in Denmark and Finland, and in 28% and 19% in Norway and Sweden, respectively. The 5-year revision rate for THAs for OA was lower in Sweden (2.3%, 95% CI 2.1–2.5) than in the Netherlands (3.0%, CI 2.9–3.1), Norway (3.8%, CI 3.6–4.0), Denmark (4.6%, CI 4.4–4.8), and Finland (4.4%, CI 4.3–4.5). Revision rates in Denmark, Norway, and Finland were higher for all fixation groups.

Interpretation — Patient and THA procedure characteristics as well as revision rates evinced some differences between the Netherlands and the Nordic countries. The Netherlands compared best with Denmark in terms of patient and procedure characteristics, but resembled Sweden more in terms of short-term revision risk. Combining data from registries like LROI and the NARA collaboration is feasible and might possibly enable tracking of potential outlier implants.

Arthroplasty registries are used to evaluate patient, procedure, prosthesis, and hospital characteristics associated with revision surgery as well as to improve quality of care (Herberts and Malchau 2000, Graves 2010). Comparison of national arthroplasty registries is important to improve our understanding of national differences and similarities. Furthermore, combining data from arthroplasty registries from several countries is needed in order to increase numbers to create the possibility of detecting inferior implants as early as possible.

The Nordic Arthroplasty Register Association (NARA) was established in 2007 by representatives from arthroplasty registries in Sweden, Norway, and Denmark to improve the quality of total hip and total knee arthroplasty through a registries-based research collaboration. Finland joined the association in 2010. To date, NARA is the most developed multinational arthroplasty database worldwide (Mäkelä et al. 2019). The comparison of national demographics and results was one of the main initial aims of the NARA collaboration. Therefore, the NARA database contains only parameters that are included in all the individual registries (Havelin et al. 2011).

The first NARA publication in 2009 described the results of 280,201 total hip arthroplasties (THAs) performed in 1995–2006 in Sweden, Norway, and Denmark (Havelin et al. 2009). This research was updated in 2014 by Mäkelä et al. using NARA data from 1995–2011 including Finland (Mäkelä et al. 2014). Substantial differences were found in the patient populations receiving a THA in the Nordic countries and in the procedure characteristics such as surgical approach and fixation (Mäkelä et al. 2014). Furthermore, substantial differences in 10-year survival rates were found between the Nordic countries (Havelin et al. 2009). The Dutch Arthroplasty Register (LROI) contains data on THAs since 2007. We initiated this study to compare patient and treatment characteristics as well as survival rates between THAs in the Netherlands and Nordic countries.

## Patients and methods

The NARA database consists of pooled data from the national hip arthroplasty registries of Denmark, Norway, Finland, and Sweden. Each register has validation routines based on national patient registries. A minimal NARA dataset was created that contains data that all registries could deliver, where personal identification numbers are deleted. The data were treated with full confidentiality, and identification of individual patients was not possible as a result of the anonymization of the NARA dataset (Havelin et al. 2009, 2011).

The degree of coverage and completeness of the Nordic registries is documented to be higher than 95% (DHAR, FAR, NAR, SHAR n.d.). Selection and transformation of the respective datasets and de-identification of the patients, including deletion of the national personal identity numbers, was performed within each national registry. Anonymous data was then merged into a common research database. Ethical approval of the study was obtained through each national registry.

The LROI is the nationwide population-based register that includes information on arthroplasties in the Netherlands since 2007. The LROI has coverage of 100% of Dutch hospitals and completeness of reporting of over 95% for primary THAs and 88% for revision arthroplasties (LROI n.d., van Steenbergen et al. 2015). The LROI database contains information on patient, procedure, and prosthesis characteristics recorded by registrars from each hospital. The LROI uses the opt-out system to require informed consent of patients.

For the present study we included all primary THAs in the period 2010–2016 registered in the NARA (n = 290,823) and the LROI (n = 184,862). Bilateral procedures were included in the study, since they do not introduce significant dependency problems in register studies (Ranstam et al. 2011). Resurfacing hip arthroplasties were excluded, though metal-on-metal total hip arthroplasties were included. Records with missing data were included.

Age was categorized into 4 groups (< 55, 55–64, 65–74, ≥ 75). The categories for diagnosis differed slightly between NARA and the LROI. We harmonized LROI variables to the NARA minimal dataset of variables, with the main harmonization for childhood diseases being developmental dysplasia of the hip, Perthes disease, and slipped capital femoral epiphysis and a combination of the latter two diagnoses in the NARA dataset compared with post-Perthes and dysplasia in the LROI (Table 1). Furthermore, surgical approach was divided into posterior and non-posterior.

**Table 1.** Harmonization of LROI variables to the NARA minimal dataset for diagnosis

NARA diagnosis	LROI diagnosis
Primary osteoarthritis	Osteoarthritis
Inflammatory arthritis	Inflammatory arthritis
Rheumatoid arthritis	Rheumatoid arthritis
Other inflammatory	
Ankylosing spondylitis	
Hip fracture	Fracture (acute)
	Late posttraumatic
Pediatric hip disease	Post-Perthes
Developmental dysplasia of the hip	Dysplasia
Perthes disease	
Slipped capital femoral epiphysis	
Combination of slipped capital	
femoral epiphysis and Perthes	
Idiopathic femoral head necrosis	Osteonecrosis
Other	Other

Cause of revision was harmonized between the NARA and the LROI. In the LROI more than one cause of revision could be registered, which is not in line with the NARA where the main reason for revision is registered. Therefore, a hierarchical order of reasons for revision (similar to the order used on the Norwegian data: top to bottom; infection, aseptic loosening, periprosthetic fracture, dislocation, other, missing) was used for the LROI data. Loosening of the acetabular and femoral component without infection as available in the LROI were combined as aseptic loosening (Table 2). In the LROI for 211 records (11%) more than one reason for revision was registered within 1 year of follow-up and 296 (8%) within the entire study period. The study is reported according to the STROBE guidelines.

**Table 2.** Harmonization of LROI variables to the NARA minimal dataset for cause of revision

NARA cause of revision	LROI cause of revision
Deep infection	Infection
Aseptic loosening	Loosening of acetabular component,
	no infection
	Loosening of femoral component,
	no infection
Periprosthetic fracture	Periprosthetic fracture
Dislocation	Dislocation
Pain only	(not available)
Others	Other
	Girdlestone situation
	Peri-articular ossification
	Inlay wear
	Symptomatic metal-on-metal bearing
Unknown	No cause of revision registered for
	revision procedure

### Statistics

Descriptive statistics of patient and procedure characteristics as well as THA incidence per year based on the population per country was calculated, according to country. Survival time was calculated as the time from primary procedure to first all-cause, any component revision, death of the patient, or end of the follow-up (January 1, 2017). Median follow-up was 3 years (interquartile range: 1.4–4.8 years). Kaplan–Meier survival analyses were performed to evaluate time to all-cause any component revision including 95% confidence intervals (CI) according to countries for OA THA patients. Stratified analyses were performed according to fixation and sex. Reasons for revision within 1 year and within 5 years’ follow-up time were described per country. For the 95% CIs, we assumed that the number of observed cases followed a Poisson distribution.

### Ethics, registration, funding and potential conflicts of interests

The dataset was processed in compliance with the regulations of the LROI and NARA governing research on registry data. No external funding was received. No competing interests were declared. 

## Results

475,685 THAs were included in the NARA and LROI databases in the study period, with 39% of procedures from the Netherlands, 24% from Sweden, and around 12% each from Denmark, Norway, and Finland (Table 3). The volume of primary THAs increased each year in all 5 countries during the study period. The 2016 incidence rates of new THAs were around 170–175 per 100,000 inhabitants in NARA countries, and 150 in the Netherlands (Table 3).

**Table 3.** Primary total hip arthroplasty volume per country

	Denmark	Norway	Sweden	Finland	The Netherlands
Annual primary THA volume					
2010	8,478	7,254	15,730	7,153	23,206
2011	8566	7,318	15,784	7,538	23,859
2012	8,536	7,822	15,958	7,895	25,299
2013	8,774	8,087	16,278	8,172	26,038
2014	9,174	8,112	16,528	8,348	28,073
2015	9,516	8,437	16,630	9,015	28,795
2016	9,913	8,873	17,261	9,673	29,592
Total	62,957	55,903	114,169	57,794	184,862
% of total	13	12	24	12	39
Population 2016 (million) and THA incidence 2016 (per 10^5^)					
Population	5.7	5.2	10.0	5.5	17.0
Incidence	174	170	173	176	150

### Patient characteristics

In the Netherlands, the proportion of patients aged < 55 years was 9%, which was lower than that in the Nordic countries; in Finland 13% of THA patients were aged < 55 years. The proportion of patients aged ≥ 75 years was comparable between the Netherlands, Denmark, and Sweden with 31–32% each, while this proportion was lower in Finland and Norway (28–29%). The proportion of male patients was lowest in the Netherlands at 34% compared with any of the Nordic countries (35–43%). The proportion of THAs due to primary osteoarthritis (OA) was higher in the Netherlands (87%) than in Sweden, Norway, and Denmark, but comparable to Finland. The proportion of hip fractures was lower in the Netherlands compared with Denmark, Sweden, and to a lesser extent Norway. The proportion of inflammatory arthritis differed between the countries, from 0.9% in the Netherlands up to 2.5% in Finland. In Denmark and Sweden hip fracture as diagnosis for THA was almost twice as frequent as in the Netherlands and almost 3 times more frequent than in Finland (Table 4).

**Table 4.** Characteristics of the total hip arthroplasty patients and procedures registered in the NARA and LROI database, 2010–2016 (n = 475,685). Values are percentages unless otherwise specified

Factor	Denmark	Norway	Sweden	Finland	The Netherlands
No. of THAs	62,957	55,903	114,169	57,794	184,862
Male sex	43	35	42	43	34
Age					
< 55	12	11	11	13	9.0
55–64	19	23	20	23	22
65–74	38	37	38	36	38
≥ 75	31	29	31	28	32
Diagnosis					
Primary osteoarthritis	81	77	81	86	87
Inflammatory arthritis	1.1	2.1	1.1	2.5	0.9
Hip fracture	11	8.2	11	3.9	6.2
Pediatric hip diseases	3.3	8.8	1.9	0.9	2.3
Idiopathic head necrosis	2.3	2.2	2.2	1.8	2.9
Others	0.9	1.3	3.0	4.8	0.7

### Procedure characteristics

Uncemented fixation of both components was used in 62% of patients in the Netherlands, in 70% of patients in Denmark and Finland, and in 28% and 19% in Norway and Sweden, respectively.

In the Netherlands, a posterior hip approach was used in 61% of the THA procedures, which was in between Sweden (52%) and Finland (67%), but lower than in Denmark (96%) and higher compared with Norway (40%) (Table 5).

**Table 5.** Procedure characteristics of the total hip arthroplasty patients and procedures registered in the NARA and LROI database, 2010–2016 (n = 475,685). Values are percentages unless otherwise specified

Factor	Denmark	Norway	Sweden	Finland	The Netherlands
No. of THAs	62,957	55,903	114,169	57,794	184,862
Fixation					
Cemented	12	31	66	11	28
Uncemented	70	28	19	71	62
Hybrid	16	3.0	2.8	15	4.6
Reverse hybrid	0.9	37	13	1.7	4.7
Unknown, uncertain	0.6	1.4	0.0	1.8	0.6
Posterior approach	96	39	52	67	^a^ 61

**^a^** For Finland approach is available since 2014, proportion shown is for 2014–2016.

### Revision

**Figure 1.**
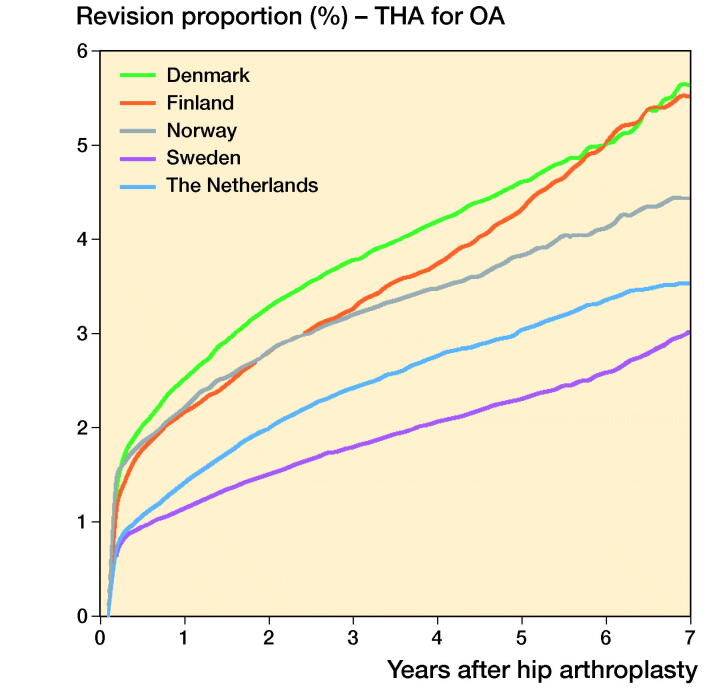
Cumulative revision proportion (%) of THAs for OA according to country, all fixation methods.

In THAs for primary OA the 1-, 3-, and 5-year overall revision rates were lowest in Sweden. The Netherlands had a higher revision rate (5-year revision rate: 3.0%, CI 2.9–3.1) compared with Sweden (2.3%, CI 2.1–2.5), but a lower revision rate compared with Denmark (4.6%, CI 4.4–4.8), Norway (3.8%, CI 3.6–4.0), and Finland (4.4%, CI 4.3–4.5) (Table 6, Figure 1). Stratified analyses showed lower revision rates for cemented THAs in Sweden and the Netherlands compared with Denmark, Norway, and Finland. Uncemented THAs had lower 3- and 5-year revision rates in the Netherlands and Sweden than in Norway, Denmark, and Finland. Hybrid (femur cemented) and reverse hybrid (cup cemented) THAs had lowest short-term revision rates in Sweden (Table 6, Figure 2). However, the number of cases with reverse hybrid THAs was small in Finland and too small in Denmark for meaningful analyses. Therefore the reverse hybrid cases from Denmark were excluded. Stratified analyses by sex showed comparable results for short-term overall revision rates examining differences between countries. Short-term overall revision rates were somewhat higher for males compared with females in all countries (data not shown). Analyses of the whole study population including cases with non-OA diagnoses gave the same results (data not shown).

Figure 2.Cumulative revision proportion (%) of cemented THAs (a), uncemented THAs (b), hybrid THAs (c), and inverse hybrid THAs (d) for OA according to country.

**Figure:**
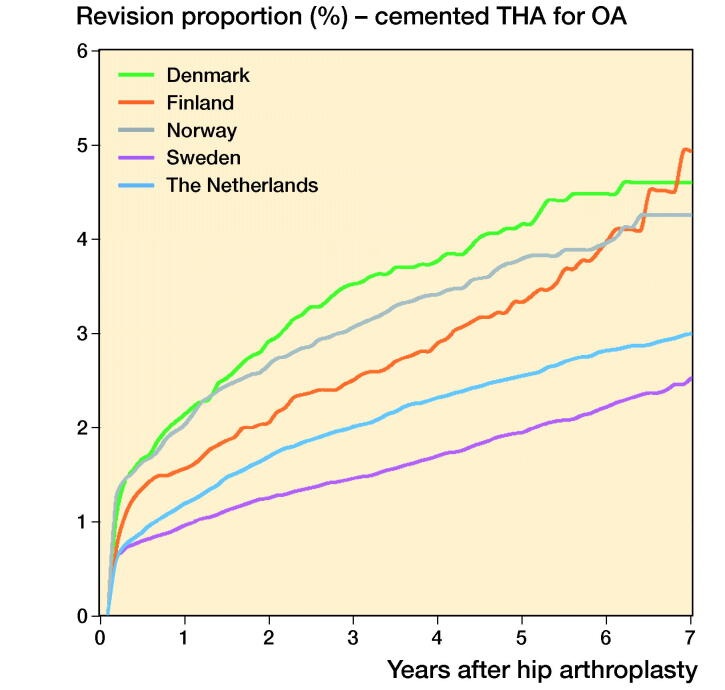

**Figure:**
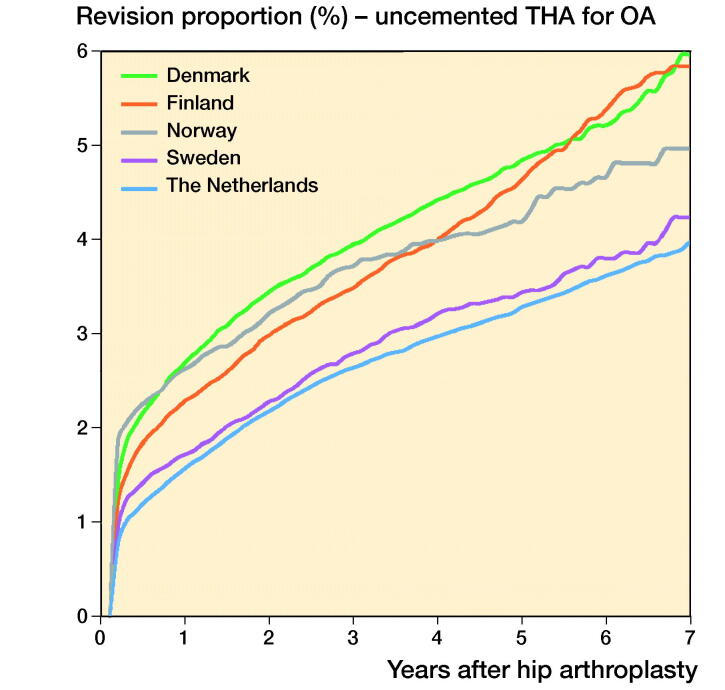

**Figure:**
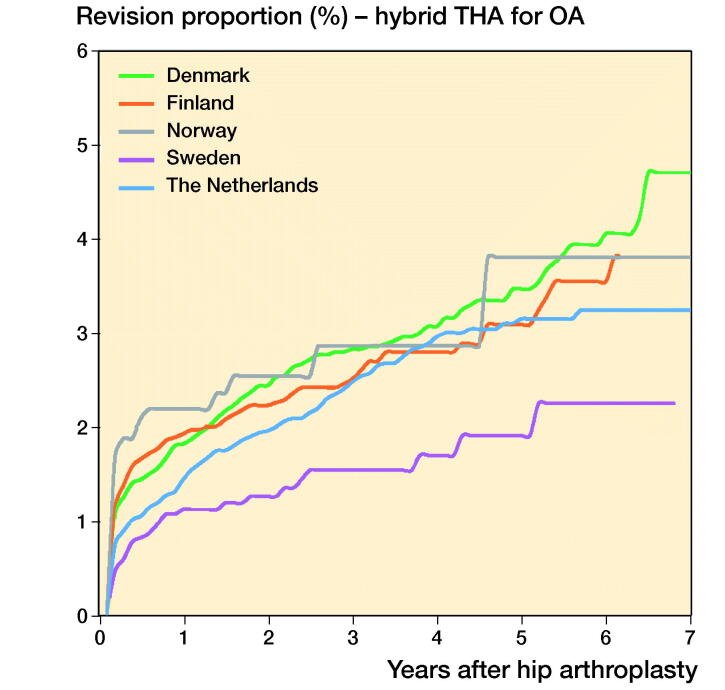

**Figure:**
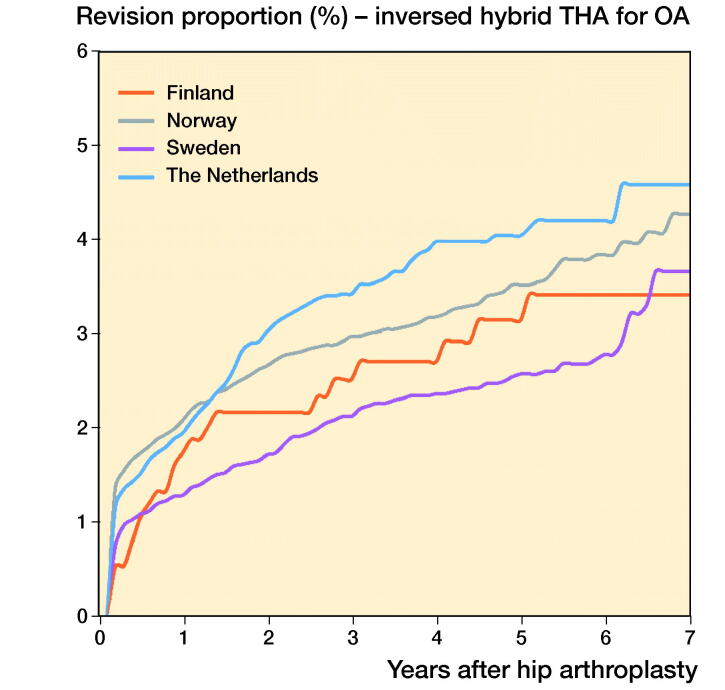

**Table 6.** Kaplan–Meier revision rates (%) with 95% confidence intervals at 1, 3, and 5 years after primary total hip arthroplasties for osteoarthritis

Factor	THAs	Revisions	1–year	3–year	5–year
Country	(n)	(n)	revision rate	revision rate	revision rate
All THAs					
Denmark	50,777	1,910	2.6 (2.4–2.8)	3.8 (3.6–4.0)	4.6 (4.4–4.8)
Norway	42,941	1,364	2.3 (2.1–2.5)	3.2 (3.0–3.4)	3.8 (3.6–4.0)
Sweden	92,069	1,722	1.2 (1.2–1.2)	1.8 (1.8–1.8)	2.3 (2.1–2.5)
Finland	48,597	1,672	2.2 (2.0–2.4)	3.3 (3.1–3.5)	4.4 (4.3–4.5)
The Netherlands	159,386	3,791	1.4 (1.4–1.5)	2.4 (2.3–2.5)	3.0 (2.9–3.1)
Cemented THA					
Denmark	5,716	202	2.2 (2.0–2.4)	3.5 (3.2–3.8)	4.2 (3.9–4.5)
Norway	13,491	423	2.1 (2.0–2.2)	3.1 (2.9–3.3)	3.8 (3.6–4.0)
Sweden	59,568	945	1.0 (1.0–1.0)	1.5 (1.4–1.6)	2.0 (1.9–2.1)
Finland	5,029	145	1.6 (1.4–1.8)	2.5 (2.3–2.7)	3.4 (3.1–3.7)
The Netherlands	43,095	765	1.0 (0.9–1.1)	1.8 (1.6–1.9)	2.2 (2.1–2.4)
Uncemented THA					
Denmark	37,330	1,478	2.8 (2.7–2.9)	4.0 (3.9–4.1)	4.9 (4.8–5.0)
Norway	11,610	415	2.7 (2.5–2.9)	3.8 (3.6–4.0)	4.3 (4.1–4.5)
Sweden	17,558	470	1.8 (1.7–1.9)	2.8 (2.7–2.9)	3.5 (3.3–3.7)
Finland	34,976	1,323	2.3 (2.2–2.4)	3.6 (3.5–3.7)	4.7 (4.6–4.8)
The Netherlands	101,249	2,608	1.5 (1.4–1.6)	2.6 (2.5–2.7)	3.3 (3.2–3.4)
Hybrid THA (femur cemented)					
Denmark	7,288	201	1.8 (1.5–2.1)	2.8 (2.4–3.3)	3.5 (2.9–4.0)
Norway	1148	28	2.2 (1.3–3.1)	2.9 (1.7–4.1)	3.8 (1.6–6.0)
Sweden	2395	34	1.1 (0.7–1.6)	1.5 (1.0–2.1)	1.9 (1.1–2.7)
Finland	7198	165	1.9 (1.6–2.3)	2.5 (2.1–2.9)	3.1 (2.5–3.7)
The Netherlands	7,242	171	1.4 (1.2–1.7)	2.5 (2.1–2.9)	3.2 (2.7–3.7)
Reverse hybrid THA (cup cemented)					
Denmark	285	21	n.a.	n.a.	n.a.
Norway	16,329	485	2.1 (1.9–2.3)	3.0 (2.7–3.3)	3.5 (3.2–3.9)
Sweden	12,546	273	1.3 (1.1–1.5)	2.1 (1.9–2.4)	2.6 (2.2–2.9)
Finland	771	22	1.7 (0.8–2.7)	2.5 (1.4–3.7)	3.1 (1.8–4.5)
The Netherlands	6,992	225	1.9 (1.6–2.3)	3.4 (3.0–3.9)	4.0 (3.5–4.6)

n.a. = not available, numbers are too small.

### Reason for revision

The most frequent reason for revision in the first year after primary THA differed between countries; dislocation was the most frequently registered reason for revision within the first year for Denmark and the Netherlands. For Norway, Sweden, and Finland deep infection was the most frequent reason for revision (Table 7). After up to 5 years of follow-up, dislocation and infection were the most common reasons for revision in the Nordic countries, while aseptic loosening was the most frequently registered reason for revision within 5 years of follow-up in the Netherlands.

**Table 7.** Reasons for revision (%) within 1 year and up to 5-year follow-up of total hip arthroplasties for osteoarthritis registered in the NARA and LROI database

Factor	Denmark	Norway	Sweden	Finland	The Netherlands
**Revisions up to the 5-year follow-up** (n = 475,685)					
THAs (n)	50,777	42,941	92,069	48,597	159,386
Revisions	1,910	1,364	1,722	1,672	3,854
Cause of revision					
Dislocation	0.98	0.55	0.36	0.64	0.60
Deep infection	0.86	1.18	0.87	0.64	0.44
Aseptic loosening	0.61	0.56	0.32	0.41	0.66
Periprosthetic fracture	0.73	0.26	0.21	0.50	0.23
Pain only	0.18	0.15	0.03	0.06	n.a.
Others	0.36	0.29	0.08	0.95	0.40
Unknown	0.03	0.18	0.00	0.25	0.10

n.a. = not available.

## Discussion

This study provides the first comparison between the NARA countries (Denmark, Finland, Norway, and Sweden) and the Netherlands for primary THA procedures. Combining data from different national arthroplasty registries like NARA and LROI is possible for patient and procedure characteristics as well as for revision rates and reasons for revision. When combining datasets some requirements are necessary such as harmonizing categories of diagnosis and reasons for revision.

### Patient and procedure characteristics

Our findings concerning patient and procedure characteristics of the NARA countries are in line with previously reported results. However, the proportion of male patients is somewhat higher in all NARA countries, especially in Norway compared with earlier NARA results (Mäkelä et al. 2014). Dutch patients who received a THA were generally somewhat older than THA patients in NARA countries, with Finland having a known younger THA population (Mäkelä et al. 2014). Furthermore, the proportion of OA as the diagnosis for primary THA was higher in the Netherlands compared with the Nordic countries, whereas the proportion of hip fractures was lower in the Netherlands compared with Denmark, Norway, and Sweden. This might be the result of the varying indications for use of THA for fracture patients in the Nordic countries and the Netherlands. The incidence of THA is in line with reported international THA incidence rates (Merx et al. 2003, Paxton et al. 2019), but seems to be somewhat lower in the Netherlands compared with the Nordic countries. This may reflect differences in demography, prevalence of various hip diseases, indications for surgical treatment, and/or healthcare policy between countries.

Apart from the differences in patient population, procedure characteristics also differed between the Netherlands and the Nordic countries. The Netherlands was most comparable to Denmark and Finland concerning THA fixation technique. These countries had a high proportion of uncemented fixation, while Norway and Sweden had a low proportion of uncemented fixation. Good results of cemented THA and inferior results of some uncemented THAs in the Swedish and Norwegian registries have encouraged the continued use of cemented THA in these countries (Havelin et al. 2009). However, uncemented fixation as well as reverse hybrid fixation increased in both Norway and Sweden during the study period.

### Revision

There were differences in short-term revision rates between NARA countries and the Netherlands for all evaluated hip stem and acetabular fixation group combinations. Although differences were small, revision risk at short-term follow-up was lowest in Sweden for cemented THAs. The Swedish Hip Arthroplasty Register (SHAR) has provided feedback and advice to the orthopedic surgeons for over 40 years (SHAR n.d.). However, Dutch results showed comparable low short-term revision rates as well. The higher short-term revision rates for THAs in Denmark and Finland might be explained by more dislocations due to the posterior approach (Zijlstra et al. 2017), which is frequently used in these countries. However, the posterior approach has been shown to give better patient-reported outcomes (Amlie et al. 2014, Rosenlund et al. 2017).

Results of large femoral head metal-on-metal THAs have been poor (Seppanen et al. 2018), which may be an important factor in the explanation for the higher revision rates in uncemented THAs in Denmark and Finland where the proportion of these THAs was high. However, in the Netherlands these large femoral head metal-on metal THAs were also used in substantial numbers (LROI n.d.). Unfortunately we were not able to exclude these big head metal-on-metal cases from our analyses because we did not include femoral head size or articulating materials in this data set. Furthermore, patient characteristics are likely to play a role in the revision rates of THAs, with Finland having a younger patient population that might partly explain their higher short-term revision rate (FAR, LROI n.d.).

Reverse hybrid and hybrid THAs had the lowest short-term revision rates in Sweden, being lower than in the Netherlands. It has been stated previously based on NARA data, however, that reverse hybrid THA has a higher revision rate than cemented THA, mainly due to periprosthetic fractures (Wangen et al. 2017). The number of reverse THAs in the Nordic countries has decreased in recent years, after the period of the current study (2010–2016).

Cause of revision differed between countries, with dislocation being the most frequent reason for revision in the Netherlands and Denmark. This is probably related to a higher proportion of the THAs done with a posterior approach. Infection represents a major share of the short-term revisions, which might substantially influence the short-term revision rate. However, infection as a reason for revision should be considered carefully, since the capture rate in arthroplasty registries is known to be suboptimal, with up to 40% of revision procedures with proven infections missing (Gundtoft et al. 2015). This might be different between countries, which most likely biases the results. Validity of revision for infection largely depends on the possibility to validate registry data with other datasets from, e.g., microbiology. Currently, a study is being performed in the Netherlands to examine the proportion of revisions for infections not registered in the LROI, which is expected to be comparable to the study by Gundtoft et al. The proportion of unknown reasons for revision differed substantially between countries, which also might bias the short-term results.

### Strengths and weaknesses

The strength of this study is the large population-based registry dataset from 5 countries with high-quality data and minimal loss to follow-up from each national register. Completeness of data in the included registries is documented to be higher than 95% (DHAR, FAR, LROI, NAR, SHAR n.d.). Furthermore, registries provide real-world data with high generalizability and external validity. This gives us the opportunity to describe trends and differences between countries. On the other hand, our registry data are observational and can be subject to bias that we cannot account for, i.e., selection bias and confounding by indication by surgeon. Therefore, causality cannot be inferred. Data used for this project is limited by being common to all 5 registries, and hence we lack information on some factors that affect the outcomes. In this first study comparing LROI and NARA data we did not merge the datasets, hampering the possibility to perform adjusted analyses.

In this study we examined and compared NARA and LROI data from a relatively recent period, resulting in a limited follow-up of THA procedures with a maximum of 7 years. Furthermore, we did not include femoral head size or type of articulation, which are known factors influencing revision rates. Moreover, we were not able to exclude metal-on-metal THAs, which have a known high revision rate (Smith et al. 2012).

In addition, we evaluated overall revision, which contains a variety of revision procedures ranging from small revision procedures like femoral head exchange to major revision procedures such as complete exchange or extraction of all components. The type of revision procedure performed might differ between countries, resulting in differences in revision rates. Moreover, the threshold for performing revision procedures may vary between countries, which influences revision rates.

Demographics of the Nordic countries and the Netherlands are comparable, although there are substantial differences in travel distance to the nearest (revision) hospital between the Netherlands and, e.g., the rural areas of the Nordic countries. Furthermore, the Nordic countries as well as the Netherlands have a solid healthcare system. However, while the health expenditure of the Nordic countries is mainly paid by the government, the health expenditure of the Netherlands is mainly covered by compulsory health insurance (OECD n.d., OECD 2019).

### Conclusion

Patient and THA procedure characteristics as well as revision rates evinced some differences between the Netherlands and the Nordic countries, although the magnitude of differences was small. The Netherlands seemed to compare best to Denmark in terms of patient and procedure characteristics, but was most comparable to Sweden in terms of short-term revision risk for both cemented and uncemented THAs. The observed absolute differences in revision risk were small and might not be clinically relevant, since they most likely are a reflection of variations in demographics, patient selection, procedure characteristics, or indications for revision. However, most of these factors cannot be studied based on the common data available. Combining data from registries like LROI and those included in the NARA collaboration is feasible. This might possibly enable tracking of potential outlier implants, specific patient groups, or events with a relatively low occurrence. Further research is warranted to merge datasets and look at more details concerning THA, creating the opportunity to perform patient-, procedure-, and prosthesis-adjusted analyses.

All authors made a substantial contribution to the study. RN, BS, and LS contributed to the conception of the study. JK created the dataset with the NARA study group. LS conducted the statistical analyses and prepared the manuscript. KM, JK, OR, SO, OV, AP, AE, GH, BS, and RN participated in the data collection and revision of the manuscript.

The authors would like to thank the technical support team at the SHAR for their help. 

**Table:** 

Factor	Denmark	Norway	Sweden	Finland	The Netherlands
**Revisions within 1 year** (n = 401,878) ^a^					
THAs	42,628	36,149	78,073	40,552	133,985
Revisions	1,420	1,087	1,338	1,145	1,935
Cause of revision					
Dislocation	0.77	0.36	0.29	0.48	0.40
Deep infection	0.71	1.08	0.77	0.54	0.27
Aseptic loosening	0.25	0.26	0.10	0.19	0.34
Periprosthetic fracture	0.61	0.21	0.17	0.41	0.18
Pain only	0.06	0.03	0.01	0.01	n.a.
Others	0.26	0.21	0.04	0.51	0.18
Unknown	0.02	0.15	0.00	0.21	0.09

**^a^** In 2010–2015 to assure at least 1-year follow-up for all records.
